# Medication-Focused Patient Counseling Upon Discharge: A Feasibility Study of Effect on Patient Satisfaction

**DOI:** 10.3390/pharmacy8010008

**Published:** 2020-01-14

**Authors:** Carina Lundby, Julia Filipsen, Susanne Rasmussen, Anton Pottegård

**Affiliations:** 1 Hospital Pharmacy Funen, Odense University Hospital, Solfaldsvej 38, Entrance 208, 5000 Odense C, Denmark; julia.filipsen@rsyd.dk (J.F.); susanne.rasmussen1@rsyd.dk (S.R.); apottegaard@health.sdu.dk (A.P.); 2 Open Patient data Explorative Network (OPEN), Odense University Hospital, J.B. Winsløws Vej 9A, 5000 Odense C, Denmark; 3 Clinical Pharmacology and Pharmacy, Department of Public Health, University of Southern Denmark, J.B. Winsløws Vej 19, 2., 5000 Odense C, Denmark

**Keywords:** patient discharge, patient satisfaction, survey, feasibility study

## Abstract

**Objective:** To develop and test a simple medication-focused patient counseling intervention at hospital discharge, with the aim of improving patient satisfaction. **Methods:** The intervention was developed during a workshop and carried out by pharmaconomists. The final intervention comprised preparing information for the discharge counseling, medication reconciliation, discussion with physician, patient counseling at discharge, medication report to primary care physician, and phone follow-up three days after discharge. The intervention was tested against usual care in a gastrointestinal surgical unit in a feasibility study, using the setup of a randomized controlled trial, with patient satisfaction as the primary outcome. **Results:** A total of 85 patients were invited to participate in the study. Following refusals (*n* = 11) and exclusions (*n* = 10), 32 patients were included in each trial arm (median age of 66.5 years; 52% males; median admission length of seven days). Patient satisfaction was high in both groups, with 75% (intervention) and 91% (control) reporting being overall satisfied with the discharge process (*p* = 0.10). No other differences between the groups were identified. **Conclusions:** The intervention did not result in improved patient satisfaction. This is likely attributed to the low number of patients included, the high satisfaction at baseline, and the lack of a validated tool to measure patient satisfaction. The developed intervention and study findings can inform future studies.

## 1. Introduction

Transitions of care, e.g., the transition from hospital to primary care following hospital discharge, is widely recognized as a vulnerable period for patients [1], inferring a high risk of medication errors and adverse drug events [2,3]. Further, following discharge, patients may feel confused about medication changes initiated during admission [4,5,6] which can lead to patients not taking their drugs as intended [3] as well as an increased rate of contact to the patients’ primary care physician or the hospital [7]. From the patients’ perspective, lack of information on medication changes prior to discharge may result in frustration [4] and a general lowering of patient satisfaction with the discharge process [5,8]. Thus, with the aim of improving patient satisfaction, we developed and tested a simple intervention based on medication-focused patient counseling at hospital discharge.

## 2. Materials and Methods

We developed a simple intervention based on medication-focused patient counseling at hospital discharge which we compared to usual care (control) in a feasibility study, with patient satisfaction as the primary outcome. The study was conducted using the setup of a randomized controlled trial, following the Consolidated Standards of Reporting Trials (CONSORT) guidelines [9].

### 2.1. Setting and Participants

The study was conducted at Odense University Hospital, a large tertiary university hospital in Denmark. Patients were recruited from a 20-bed gastrointestinal unit within the surgical department. Patients admitted to this unit are usually treated for inflammatory bowel disease, cancer within the gastrointestinal system, or complex fistulas.

Based on preliminary data for the number of patients discharged per week, a study period of five months (from October 2017 through February 2018) was chosen with the aim of including 50 patients in each trial arm. Due to the lack of a standardized measure for patient satisfaction, no formal power calculation was performed. Patients were eligible if they were ≥18 years, spoke Danish, were not cognitively impaired, and were discharged from the gastrointestinal unit. Patients were randomly assigned to the intervention or usual care in a 1:1 ratio using block randomization (blocks of 4 and 6). The software Research Electronic Data Capture (REDCap) was used for randomization as well as storage of the collected data [10].

### 2.2. Intervention

Initially, a broad outline of the intervention was proposed based on the experiences of two pharmaconomists (J.F. and S.R.) working within the gastrointestinal unit, both recognizing the need for more involvement of patients on changes to their medical treatment. Pharmaconomists are comparable to pharmacy technicians, although they have a substantially longer education of three years, and are authorized to dispense and check prescriptions as well as to counsel patients about use of medication [11]. Hereafter, the details of the intervention were composed through a workshop with participation of pharmaconomists, clinical pharmacists, primary care pharmacists, and health care researchers, including a researcher from the gastrointestinal unit. This workshop aimed to explore how health care professionals from primary as well as secondary care experience and handle problems related to patients’ lack of knowledge on medication changes following discharge.

**Figure 1:**
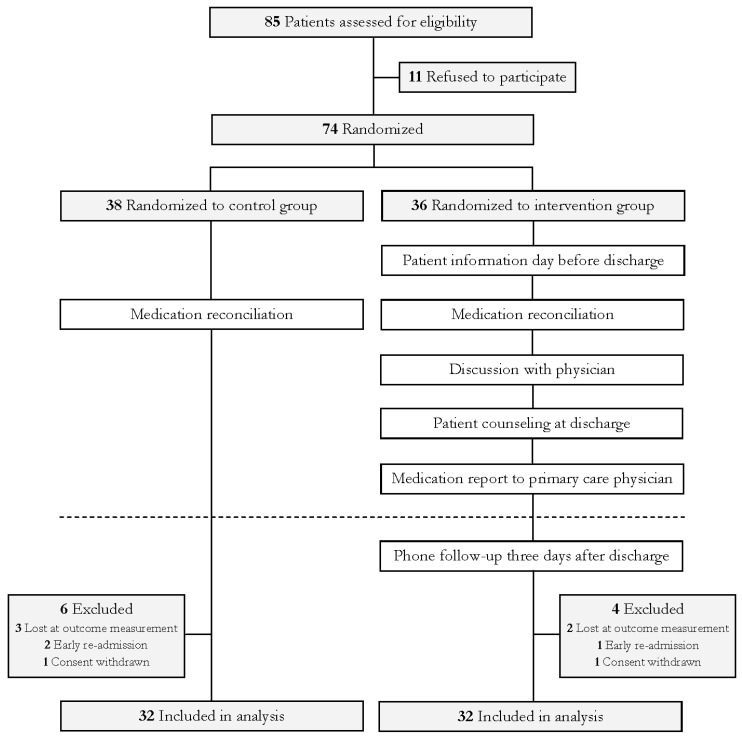
Flowchart detailing the intervention.

The final intervention (Figure 1) comprised patient information the day before discharge (preparing information for the discharge counseling), medication reconciliation, discussion with physician, patient counseling at discharge, medication report to primary care physician, and phone follow-up to the patient three days after discharge. The discharge counseling included an updated medication list and a written summary of the counseling for the patient to take home, including a direct phone number to the pharmaconomist performing the counseling. The intervention was pilot tested and refined at another gastrointestinal surgical unit for ten weeks prior to the study. Usual care consisted of a medication reconciliation (Figure 1) as well as usual discharge procedures within the surgical department. Patient recruitment and the intervention were carried out by two pharmaconomists (J.F. and S.R.). A full outline of the intervention is provided in the Supplementary Materials.

### 2.3. Outcome Measures

The primary outcome was patient satisfaction, including perceptions of transition of care, measured by a six-item Likert scale questionnaire. This outcome was chosen based on input from the surgical department. Answers were obtained seven days after discharge via a telephone survey by a research pharmacist blinded to group allocation. The pharmacist was located at the hospital pharmacy and had no connection to either the clinic or specific department. The pharmacist only received the patient’s name and phone number prior to the survey. The questionnaire was based on a validated questionnaire used in an annual national survey on patient experiences of being admitted to Danish public hospitals [12]. The six questions were followed by an open-ended question, giving the patients an opportunity to elaborate their answers. The questionnaire was pilot tested and refined along with the intervention.

As a supplement to the quantitative outcome measure, a qualitative study was mounted to explore patients’ experiences based on individual semi-structured interviews with five patients from each trial arm. These data have been reported in a separate paper [13].

### 2.4. Data Analysis

Data were analyzed according to intention to treat. Characteristics of study participants were analyzed using descriptive statistics. Differences in the outcome measure (patient satisfaction) were compared between the intervention and control group using a chi-squared test (95% level of significance). Data analysis was performed using STATA IC v15.1 (StataCorp).

### 2.5. Ethical Considerations

The study was conducted in accordance with the Declaration of Helsinki. The study was approved by the Danish Data Protection Agency (approval 17/18948), while the Regional Committees on Health Research Ethics waived registration.

## 3. Results

A total of 85 patients were invited to participate in the study of whom 11 refused. Of the remaining 74 patients, 36 were randomized to the intervention group while 38 were randomized to the control group. Following dropouts (withdrawal of consent (*n* = 2), early re-admission (*n* = 3), and no answering of phone for the outcome measurement (*n* = 5)), each trial arm included 32 patients. The patients had a median age of 66.5 years (interquartile range (IQR), 54–72) and 33 of the 64 patients were male. The patients had been admitted for a median of seven days (IQR, 5–11.5) before receiving the discharge intervention. In the period where 85 patients were invited to participate, another 224 patients passed through the department. The reasons for failing to invite these patients into the study were patients being discharged during weekends or when the pharmaconomists were not available (*n* = 156), patients not meeting the inclusion criteria (*n* = 33), patients being transferred to other departments or hospitals (*n* = 26), and other reasons (*n* = 9).

For the intervention patients, the patient information provided the day before discharge was only successfully applied for 19% (6/32) while the phone follow-up three days after discharge was completed for 91% (29/32). The remaining elements of the intervention were all successfully applied for all patients. Finally, following discharge, 13% (4/32) made use of the direct number to the pharmaconomists with questions regarding their medical treatment. The mean time spent on providing the full intervention, including the phone follow-up, was 32 min (range, 25–45 min).

**Figure 2:**
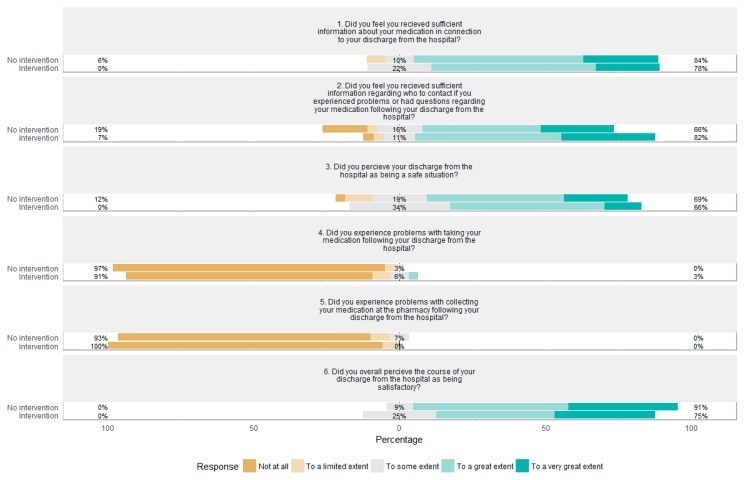
Diverging stacked bar chart displaying the distribution of answers to the six Likert scale questions, asked through the telephone survey seven days after discharge, for the intervention and control group. Note that questions 1, 2, 3, and 6 were asked so that agreement indicated satisfaction with the discharge process, while for question 4 and 5, disagreement indicated satisfaction.

Patient satisfaction was generally very high in both groups, with 75% (24/32) and 91% (29/32) answering “to a great extent” or “to a very great extent” to being overall satisfied with their course of discharge in the intervention and control group, respectively (*p* = 0.10). Similar patterns were also observed for the remaining five questions (Figure 2), with no differences achieving statistical significance. During the open-ended question, many intervention patients expressed satisfaction with having received a direct number to the pharmaconomists, as it made them feel safe knowing that they could always reach a health care professional with questions regarding their medical treatment. This was, however, not recorded systematically. During the telephone surveys, group allocation was compromised by four patients (two patients from each group).

## 4. Discussion

In this study, we developed a simple intervention to be delivered by pharmaconomists aimed at improving patient satisfaction upon discharge from the hospital. The intervention was tested in a feasibility study using the setup of a randomized controlled trial but failed to show any improvements in patient satisfaction.

The main strengths of the study are the development of a tailored intervention and the use of a randomized controlled design. The principle weakness of the study is the limited statistical power. Due to the lack of a validated and specific tool to assess patient satisfaction as well as a standardized measure for patient satisfaction, it was not possible to perform a formal power calculation. Coupled with the high satisfaction at baseline, the study had insufficient power to detect an effect of the intervention. However, although it was not possible to detect an effect, it is also possible that a larger number of patients might have confirmed the apparent results (that is, a potential negative effect of the intervention). Further, only 19% of the intervention patients received the preparing information for the counseling the day before discharge. This was primarily due to uncertainty regarding the time of discharge but also related to the fact that some patients were first included in the study at the day of discharge. As only close to one fifth of the patients received the full intervention, this might have affected the results negatively. Despite these limitations, we believe that our results provide valuable insights into patients’ perceptions of transition of care.

The qualitative study conducted as a supplement to this study also revealed that the intervention did not improve the patients’ experiences of information about medication. However, it found that the patients experienced a series of encounters with unfamiliar health care professionals which resulted in patients not requesting the necessary information about medications and treatment plans [13].

Although a significant number of studies have measured the effects of patient counseling upon discharge [14], we have only identified a few previous studies comparable to ours. Sarangarm et al. [15] reported a large randomized trial (*n* = 279) testing the effects of pharmacist-based discharge counseling, covering medication administration, side effects, and disease state education. While no effect was seen on the rate of re-admissions, patient satisfaction (measured by a nine-item survey with a five-point Likert response scale) was improved in the intervention with a mean score of 43.1 (of 45) compared to 40.4 in the control arm (*p* < 0.001). However, there was a markedly different response rate between the two groups (69% vs. 54%), raising the possibility of reporting bias. Cawthon et al. [16] reported that patients randomized to receive the intervention in the Pharmacist Intervention for Low Literacy in Cardiovascular Disease (PILL-CVD) study [17] found it helpful to speak to a pharmacist about their medication before being discharged as well as receiving a phone follow-up after discharge.

Based on the above-mentioned limitations to the present study, a refined intervention should be tested on a larger scale in a setting where there is more room for improvement from baseline regarding patient satisfaction. Before doing so, however, it is imperative to identify or develop a more specific tool to assess patient satisfaction.

## 5. Conclusions

We failed to identify any improvement in patient satisfaction from implementing a pharmaconomist-based discharge counseling service in this feasibility study. Several reasons, however, affected the study findings that could explain the lack of an effect, most importantly limited statistical power and a high patient satisfaction at baseline. Future studies could use the present findings as a starting point.

## Supplementary Materials

The following are available online at https://www.mdpi.com/2226-4787/8/1/8/s1.
