# Supplementary material for: Medication-Focused Patient Counseling Upon Discharge: A Feasibility Study of Effect on Patient Satisfaction
Source: Pharmacy (Basel). 2020 Jan 14;8(1):8. doi: 10.3390/pharmacy8010008 (PMC7151701; doi:10.3390/pharmacy8010008)
Supplement: Supplementary file 1 [file pharmacy-08-00008-s001.pdf]

Authors: Carina Lundby, Julia Filipsen, Susanne Rasmussen, and Anton Pottegård
Corresponding author: Carina Lundby, Hospital Pharmacy Funen, Odense University Hospital, carina.lundby.olesen@rsyd.dk

The intervention

The final intervention comprised patient information the day before discharge (preparing information for the discharge counseling), medication reconciliation, discussion with physician, patient counseling at discharge, medication report to primary care physician, and phone follow-up to patient three days after discharge. In the following, each part of the intervention is described in detail.

Patient information the day before discharge

The day before discharge, the pharmaconomist visited and informed the patient about the planned discharge counseling. The pharmaconomist provided the patient with a short leaflet explaining the purpose and course of the counseling. The pharmaconomist explained that the counseling would be based on the patient's needs and encouraged the patient to think about any questions he/she might have regarding medication, illness, or other concerns about being discharged. The leaflet included a few lines for the patient to write down questions.

Medication reconciliation

Prior to the discharge, the pharmaconomist conducted a medication reconciliation. When necessary, the pharmaconomist consulted a clinical pharmacist. Based on the medication reconciliation, the pharmaconomist prepared a draft version of the patient's discharge report.

Discussion with responsible physician

Based on the medication reconciliation and the prepared discharge report, the pharmaconomist consulted the physician responsible for the patient. If the pharmaconomist had found any discrepancies that could not be solved by consulting a clinical pharmacist, the pharmaconomist presented and discussed these with the physician until a clarification was reached. Further, the pharmaconomist made sure that the physician had prepared prescriptions on any new medications, removed prescriptions for discontinued medications, and, when relevant, ensured correct reimbursement for new prescription medications.

Patient counseling at discharge

Based on the medication reconciliation and physician counseling, the pharmaconomist conducted a 15 minute discharge counseling with the patient. If the patient had prepared questions or expressed any needs for discussing specific topics, these were prioritized. Otherwise, the pharmaconomist went

through the patient's medication list and explained the reasons for any medication changes, such as changed doses, new medications, and discontinued medications. Further, when relevant, the pharmaconomist covered items such as drug administration, treatment duration, and specific side effects. At the end of the counseling, the pharmaconomist provided the patient with the updated medication list and a written summary of the counseling for the patient to take with him/her back home. The summary included a direct phone number to the pharmaconomist in case the patient should think of any questions regarding his/her medical treatment following discharge. If the patient was discharged prior to a weekend, the pharmaconomist provided the patient with new medication for 2-3 days, until the patient had the opportunity to pick of the medication at a community pharmacy.

Medication report to primary care physician

Following the patient counseling, the pharmaconomist finished the patient's discharge report and made a separate medication report to the patient's primary care physician. The medication report included a short description of the patient's current medication and any medication adjustments made during hospitalization.

Phone follow-up to patient three days after discharge

Three days after discharge, the pharmaconomist made a phone follow-up to the patient. The pharmaconomist asked if the patient had experienced any medication-related problems or had any questions concerning his/her medication, giving the patient the opportunity to discuss these with the pharmaconomist.
